# Clinical and Spectral-Domain Optical Coherence Tomography Findings of Patients with Incomplete Visual Recovery after Anatomically Successful Retinal Detachment Surgery

**DOI:** 10.1155/2015/420401

**Published:** 2015-02-16

**Authors:** Miltiadis K. Tsilimbaris, Aikaterini Chalkia, Chrysanthi Tsika, Anastasios Anastasakis, Georgios A. Kontadakis

**Affiliations:** Department of Ophthalmology, University Hospital of Heraklion, University of Crete, 71003 Heraklion, Greece

## Abstract

*Purpose*. To identify causes of incomplete visual recovery in patients with anatomically successful retinal detachment surgery. *Methods*. This was a retrospective study of 61 eyes of 61 patients with at least 12-month follow-up and complete preoperative, intraoperative, and postoperative record. Postoperative visual acuity (VA) more than 0.18 logMAR was considered as incomplete visual recovery. Complete ophthalmic examination and Spectral-Domain OCT (SD-OCT) imaging were performed at last follow-up. *Results*. Twenty-nine eyes (47.5%) had a postoperative VA < 0.18 logMAR and 32 eyes (52.5%) had a postoperative VA ≥ 0.18 logMAR. Mean follow-up was 32.8 ± 17.3 months. Incomplete visual recovery was strongly correlated with presence of macular pathology (*P* = 0.002), a detached macula preoperatively (*P* = 0.02), retinotomy (*P* = 0.025), and pars plana vitrectomy and use of silicon oil as a tamponade agent (*P* = 0.009). Also, although there was a strong correlation between ellipsoid zone disruption and incomplete visual recovery, a distinct, more course pathology could be identified in all cases of poor visual recovery related to edema, thickening, or atrophy of the macula. *Conclusion*. The careful postoperative evaluation of the macula using biomicroscopy and SD-OCT can help in diagnosis of alterations that can be associated with incomplete visual recovery.

## 1. Introduction

Advances in vitreoretinal surgical techniques during the last 30 years resulted in high percentages of anatomical success in rhegmatogenous retinal detachment (RRD) repair surgery. Despite the high percentages of satisfactory anatomic results, very often the functional results still remain puzzling.

Pre- and postoperative factors have been studied in order to identify those that could potentially affect the postoperative functional outcome. The preoperative visual acuity, the time before surgical intervention, the preoperative status of the macula, the height of detachment, and the presence of vitreomacular traction have been correlated with the final visual acuity [1, 2]. In addition, postoperative macular abnormalities after anatomically successful RRD surgery have been associated with incomplete visual recovery [2–4]. Macular abnormalities such as cystoid macular edema (CME), epiretinal membranes (ERM), pigment alterations, or a macular hole can occur after anatomically successful repair and lead to incomplete visual recovery. The presence of subretinal fluid (SRF), not always clinically evident, may be also associated with poor visual outcome.

The recent development of Spectral-Domain Optical Coherence Tomography (SD-OCT) provided an additional tool for clinicians for in vivo studies of the individual layers of the macula with high resolution and thus provided further information for structural postoperative macular changes. Most of the studies utilizing the SD-OCT emphasize the value of the integrity of the photoreceptors layers as an index for visual rehabilitation after anatomically successful retinal detachment surgery. However, discrepancies between the results of the different studies still exist [1, 5–8].

The purpose of this study was to identify the causes of incomplete visual recovery in patients with anatomically successful retinal detachment surgery in an attempt to further address the existing discrepancy between anatomical and functional results.

## 2. Methods

We retrospectively reviewed the medical records of patients that underwent anatomically successful repair of RRD at the Department of Ophthalmology in the University Hospital of Heraklion, Crete. Patients were included in the study only if they had a follow-up period of at least 12 months and if a complete preoperative, intraoperative, and postoperative record was available. All surgeries had been performed by one of the authors (MKT). Preoperative data collection included a complete medical and ophthalmic history, preoperative corrected distance visual acuity (CDVA), intraocular pressure, lens status, and status of the macula at the time of presentation (macula on or off). Postoperative follow-up included a complete ophthalmic examination with measurement of the BCVA and intraocular pressure, slit lamp examination, and fundus biomicroscopy. Patients with preexisting macular disease, a history of amblyopia, retinal detachments due to giant tears or trauma, vitreous hemorrhages, or diabetes mellitus were excluded from our study.

A total of 61 eyes of 61 patients fulfilled the inclusion criteria. Included patients were divided into 2 groups according to the postoperative VA; group A, patients with visual acuity < 0.18 logMAR, and group B, patients with visual acuity ≥ 0.18 logMAR. Visual acuity of 0.18 logMAR was arbitrarily chosen as a cut-off value in order to include patients with satisfactory visual recovery in group A. Postoperative VA ≥ 0.18 was defined as incomplete visual recovery for the purpose of our study. All patients from both groups were invited to participate in an additional ophthalmic examination in an attempt to explain the postoperative visual outcome.

Additional ophthalmic examination comprised CDVA, slit lamp examination of the anterior segment, fundus biomicroscopy, and Spectral-Domain OCT (SD-OCT) imaging (Spectralis OCT, Heidelberg Engineering, Heidelberg, Germany). SD-OCT scan was used in the evaluation of the mean central foveal thickness (CFT) and in the study of the photoreceptors layers. Images were acquired using horizontal raster pattern scans, which were obtained via a 20 × 20 degree (5.4 × 5.4) scan field, consisting of 49 sections. The study was approved by the institutional review board of the University of Crete and all participants signed an informed consent form.

### 2.1. Statistical Analysis

For statistical analysis, the SPSS Statistics version 19 program was used. Visual acuity was recorded using the Standard Snellen eye Charts and was converted to the minimum angle resolution (logMAR) units for our data analysis. Comparisons between the groups were performed with the independent samples *t*-test for continuous variables and with the chi-squared test or the Fisher exact test for dichotomous data. The Spearman rank correlation coefficient was used as a measure of association between nonnormally distributed variables. *P* values ≤0.05 were considered to be statistically significant.

## 3. Results

Mean age of our patients was 63 years (range 17–82). Thirty-three eyes (54%) had a macula-on RRD and 28 eyes (46%) had a macula-off RRD at presentation. Thirty patients were phakic and thirty-one were pseudophakic. Forty-six eyes (75.4%) achieved anatomic success with 1 surgery, 11 eyes (18%) required 2 surgeries, and 4 eyes (6.6%) required 3 surgeries. In total, pneumatic retinopexy (PR) was performed in 28 eyes, scleral buckling (SB) in 14 eyes (23%), pars plana vitrectomy (PPV) and use of SF6 as a tamponade agent in 18 eyes (29.5%), and pars plana vitrectomy and use of silicone oil as a tamponade agent in 16 eyes (26.2%). Six eyes received retinectomy and 15 also underwent cataract surgery either as a combined or as a separate procedure. Duration of macular detachment prior to surgery had not been possible to be accurately estimated for all our patients and it was not included in our data analysis.

Twenty-nine eyes (47.5%) had a postoperative VA < 0.18 logMAR (group A). Reevaluation of these eyes revealed no pathological findings in 19 patients (65.5%), presence of ERM in 9 patients (31%) with no significant alteration of the CFT in comparison to the rest of the patients in this group (mean CFT in ERM patients 301 ± 95 *μ*m, *P* = 0.779), and subretinal fluid in 1 patient (3.5%) (CFT = 257 *μ*m). Only one patient presented disruption of the ellipsoid zone.

**Figure 1:**
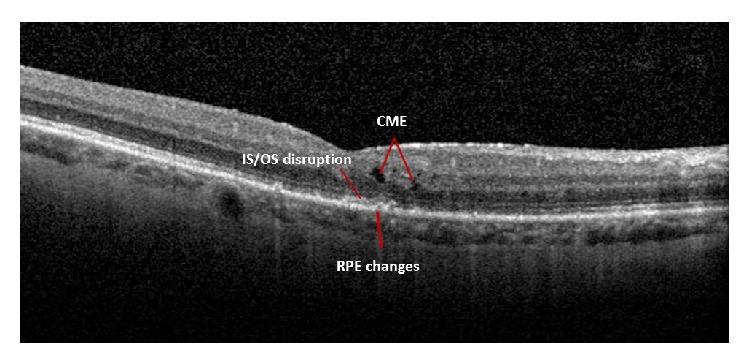
SD-OCT image of a 63-year-old patient one year after anatomical repair of a macula-on RRD with 3PPV + SF6. We can notice the presence of CME, IS/OS (ellipsoid zone) disruption, and RPE changes.

Thirty-two eyes (52.5%) had a postoperative VA ≥ 0.18 logMAR (group B). Findings from the reevaluation of this group included anterior segment pathology in 9 eyes (28%) (8 eyes developed cataract and 1 bullous keratopathy), CME in 5 eyes (16%) (mean CFT = 358 ± 83 *μ*m), ERM in 16 eyes (50%) with significant alteration of the CFT (mean CFT = 471 ± 163 *μ*m, *P* = 0.001), macular atrophic changes in 1 eye (3%) (CFT = 261 *μ*m), and a macular hole in 2 eyes (6%). Sixteen patients of group B (50%) presented with disruption of the ellipsoid zone and 12 (75%) of them had a CDVA ≥ 0.5 logMAR (*P* = 0.004) (Figure 1).

There were statistically significant differences in the percentage of patients with anterior segment pathology and macular pathology between the groups. In addition, significantly more patients in group B had presented with macula-off detachment than in group A. Regarding SD-OCT findings, CFT was significantly larger in group B than group A, and percentage of patients with ellipsoid zone disruption also was significantly higher in group B. Comparisons of preoperative and postoperative values between groups are presented in Table 1.

Furthermore, incomplete visual acuity was strongly correlated with the presence of macular pathology (*P* = 0.002), with the presence of a detached macula (macula-off retinal detachment) preoperatively (*P* = 0.02), with retinotomy (*P* = 0.025), and with pars plana vitrectomy and use of silicon oil as a tamponade agent (*P* = 0.009). No correlation was found between the number of surgeries (*P* = 0.13) and the visual outcome.

The study of the integrity of the photoreceptors layers revealed a strong correlation between ellipsoid zone disruption and incomplete visual recovery (*P* = 0.003). Ellipsoid zone disruption was also strongly correlated with the number of surgeries (*P* = 0.01), retinotomy (*P* = 0.009), and the presence of other macular pathology postoperatively (*P* = 0.01).

Since the preoperative status of the macula is an already well recognized prognostic factor for the visual outcome, we performed a subgroup analysis of the patients with preoperative macula-on detachment. According to the results, among the patients with macula-on detachment, in group A 42% had macular pathology and in group B 77% had macular pathology (*P* = 0.03, chi-squared test). Also, in group A 100% of patients had normal ellipsoid zone in SD-OCT and in group B 46% of patients had disruption of ellipsoid zone (*P* = 0.00014, chi-squared test). Average CFT was 284.42 (standard error 16.46) in group A and 378.61 (standard error 38.18) in group B (*P* = 0.017, independent samples *t*-test).

## 4. Discussion

Incomplete visual recovery can occur after anatomically successful repair of rhegmatogenous retinal detachment. The findings of our study indicate that anatomical changes at the level of the macula are in most of the cases the reason of poor visual recovery after a successful retinal detachment surgery. Although the evaluation of microanatomy of the macula (photoreceptors layer) may be helpful for further understanding the pathophysiology of poor vision, in our group of patients incomplete visual recovery in the absence of anterior segment pathology was always associated with macroanatomical features (edema, thickening, or atrophy of the macula) that could explain the situation, even in patients with preoperatively attached macula.

Cystoid macular edema appears to be the most frequent postoperative macular complication associated with poor visual outcome. However, CME tends to decrease with time and sometimes disappear spontaneously. A cataract extraction surgery before or in combination with the RRD surgery and a macular detachment may be associated with the presence of CME postoperatively according to previous studies [4, 9]. In our study, only 5 eyes of group B (16%) presented with CME. Their low number could be explained by the fact that all our patients had a follow-up of at least 12 months. No correlation was observed between the presence of CME and the cataract extraction surgery but this could be due to the low number of cases.

Epiretinal membrane formation resulting in contraction and distortion of the retina is another complication that can be associated with decreased visual acuity and metamorphopsia after anatomically successful retinal detachment surgery [5, 10]. In the present study, we evaluated the presence of ERM with SD-OCT imaging and the macula distortion caused by the ERM with the measurement of CFT. The presence of ERM was quite frequent in both groups (nine patients of group A, 31%, and 16 patients of group B, 50%) but only the 16 patients with incomplete visual recovery of group B presented with significant alterations of the CFT due to the ERM. The mean CFT of patients with ERM in group B was 471 ± 163 *μ*m. It seems that presence of ERM in OCT without a significant alteration in foveal thickness does not necessarily predetermine a low visual acuity.

Another less frequent pathology related to poor visual outcome is the macular atrophic changes and the development of a macular hole during or immediately after RRD surgery [11]. Our data analysis suggests that three such patients were found in the group with low visual acuity after RRD repair. One patient with macular atrophic changes (3%) and 2 patients (6%) with a macular hole were found in group B.

The presence of residual subretinal fluid, not clinically evident or undetectable by fluorescein angiography, but identified on OCT scans, can be also associated with poor visual outcome [3, 12]. Desatnik et al. reported that delayed subretinal fluid absorption is uncommon after pneumatic retinopexy [13]. Benson and associates report that persistent SRF after vitrectomy and gas endotamponade surgery for retinal detachment can occur; however its incidence seems to be less than that after scleral buckle surgery (15% compared with 55%). However, this may be related to the different retinal detachment and patient characteristics or may be secondary to the difference in the two surgical approaches [14]. Seo et al. show that the presence and extent of persistent submacular fluid after successful scleral buckle surgery for acute macula-off RRD may delay visual recovery but do not influence final VA or anatomic attachment [15]. In our study, subretinal fluid was observed in only 1 patient of group A (3.5%) (CFT = 257 *μ*m). The patient had undergone pneumatic retinopexy and had a follow-up of twelve months.

Patients undergoing RRD repair surgery can also present with impaired postoperative visual acuity in the absence of any evident postoperative complications. A recent study by Wakabayashi and associates where SD-OCT evaluated microstructural changes that may influence the visual outcome in such patients noted a disrupted ellipsoid zone in 23 of 38 preoperatively macula-off eyes after anatomically successful retinal detachment surgery and found that the integrity of both the ELM and the ellipsoid zone was significantly associated with better visual outcome (almost equal to that of the eyes with a preoperative macula-on RD) [5]. According to Lai et al. [6] the presence of 1 or more abnormalities among the ELM, the ellipsoid zone, or the interdigitation zone postoperatively is correlated with poor postoperative BCVA, whereas Delolme et al. [7] show that in their study final visual acuity did not differ between patients with simultaneous ellipsoid zone and ELM disruptions and patients with ellipsoid zone disruption and intact ELM and that postoperative ellipsoid zone lesions were not significantly correlated with BCVA but with low macular sensitivity and thinner photoreceptor outer segment layer. In our study sixteen patients of group B (50%) presented with disruption of the ellipsoid zone (*P* = 0.003) and 12 (75%) of them had a CDVA ≥ 0.5 logMAR (*P* = 0.004). All these patients however had at the same time macroanatomical alterations that were visible in biomicroscopy or OCT imaging.

## 5. Conclusion

To conclude, our study offered an insight into the reasons of low visual acuity after successful repair of macular detachment. The main limitations of this study are its retrospective design, its relative small sample, varying intervals of postoperative SD-OCT examination, and the lack of preoperative imaging of the macula with SD-OCT. In summary, the findings of our study indicate that the postoperative status of the macula is critical for the visual outcome and thus the careful postoperative evaluation of the macula using biomicroscopy and SD-OCT can help in diagnosis of alterations that can be associated with incomplete visual recovery.

## Figures and Tables

**Table 1:** Preoperative and postoperative parameters and comparisons between groups.

*n* (%)	Total (61)	Group A (29)	Group B (32)	*P* value
	Mean (SD)			
Age	63 (11.1)	61 (11.2)	66 (10.6)	0.09^*^
Preop. CDVA	1.1 (1.08)	0.75 (0.93)	1.4 (1.1)	0.015^*^
Postop. CDVA	0.38 (0.52)	0.07 (0.12)	0.63 (0.54)	<0.0001^*^
Follow-up	33 (17)	31 (19)	36 (16)	0.46^*^
Postop. CFT	353.7 (136.1)	283 (66.6)	419.4 (156.9)	0.0002^*^

	*n* (%)			
Macula off	28 (46)	9 (31)	19 (59)	0.03^†^
Anterior segment pathology	9 (15)	0	9 (28)	<0.0001^†^
Macular pathology	34 (56)	10 (34)	24 (75)	0.002^†^
ERM	25 (41)	9 (31)	16 (50)	0.12^†^
CME	5 (8)	0	5 (16)	
other	4 (7)	1 (3)	3 (9)	
Ellipsoid zone disruption	17 (28)	1 (3)	16 (50)	<0.0001^†^

CDVA: corrected distance visual acuity, CFT: central foveal thickness, ERM: epiretinal membrane, and CME: cystoid macular edema.

^*^ *P* value derived from independent samples *t*-test.

^†^ *P* value derived from chi-squared test.
